# Spatial and temporal distribution of lumpy skin disease outbreaks in Uganda (2002–2016)

**DOI:** 10.1186/s12917-018-1503-3

**Published:** 2018-06-01

**Authors:** Sylvester Ochwo, Kimberly VanderWaal, Anna Munsey, Christian Ndekezi, Robert Mwebe, Anna Rose Ademun Okurut, Noelina Nantima, Frank Norbert Mwiine

**Affiliations:** 1 College of Veterinary Medicine, Animal resources and Biosecurity, Makerere University, P.O.BOX 7062 Kampala, Uganda; 2 College of Veterinary Medicine, University of Minnesota, 1365 Gortner Avenue, St. Paul, MN 55108 USA; 3 Ministry of Agriculture Animal Industry & Fisheries, Berkley Ln, Entebbe, Uganda

**Keywords:** Lumpy skin disease, Epidemiology, Agro-ecological zones, Spatio-temporal epidemiology, Uganda

## Abstract

**Background:**

Lumpy skin disease (LSD) is a devastating transboundary viral disease of cattle which causes significant loss in production. Although this disease has been reported in Uganda and throughout East Africa, there is almost no information about its epidemiology, spatial or spatio-temporal distribution. We carried out a retrospective study on the epidemiology of LSD in Uganda between the years 2002 and 2016, using data on reported outbreaks collected monthly by the central government veterinary administration. Descriptive statistics were computed on frequency of outbreaks, number of cases, vaccinations and deaths. We evaluated differences in the number of reported outbreaks across different regions (agro-ecological zones), districts, months and years. Spatial, temporal and space-time scan statistics were used to identify possible epidemiological clusters of LSD outbreaks.

**Results:**

A total of 1161 outbreaks and 319,355 cases of LSD were reported from 55 out of 56 districts of Uganda. There was a significant difference in incidence between years (*P* = 0.007) and across different regions. However, there was no significant difference in the number of outbreaks per month (*P* = 0.443). The Central region reported the highest number of outbreaks (*n* = 418, 36%) followed by Eastern (*n* = 372, 32%), Southwestern (*n* = 140, 12%), Northern (*n* = 131, 11%), Northeastern (*n =* 37, 3%), Western (*n =* 41, 4%) and Northwestern (*n* = 22, 2%) regions. Several endemic hotspots for the circulation of LSD were identified in the Central and Eastern regions using spatial cluster analyses. Outbreaks in endemic hotspots were less seasonal and had strikingly lower mortality and case-fatality rates than the other regions, suggesting an underlying difference in the epidemiology and impact of LSD in these different zones.

**Conclusion:**

Lumpy Skin disease is endemic in Uganda, with outbreaks occurring annually in all regions of the country. We identified potential spatial hotspots for LSD outbreaks, underlining the need for risk-based surveillance to establish the actual disease prevalence and risk factors for disease maintenance. Space-time analysis revealed that sporadic LSD outbreaks tend to occur both within and outside of endemic areas. The findings from this study will be used as a baseline for further epidemiological studies for the development of sustainable programmes towards the control of LSD in Uganda.

**Electronic supplementary material:**

The online version of this article (10.1186/s12917-018-1503-3) contains supplementary material, which is available to authorized users.

## Background

Lumpy skin disease (LSD) is an acute to sub-acute viral disease of cattle that is defined by fever, increased nasal secretions, enlarged lymph nodes, formation of nodules on the skin, mucous membranes and internal organs, edema of the skin and sometimes death [1–3]. Lumpy skin disease virus (LSDV) is classified within the genus Capripoxvirus in the family Poxviridae, which includes the closely related viruses of sheep pox and goat pox [2]. Although cattle are the natural host of LSDV, clinical infection has been observed in the Asian water buffalo from Egypt [4] and antibodies have been reported in black and blue wildebeest, eland, giraffe, greater kudu, African buffalo, and other animal species [5, 6]. LSDV neither infects nor is it transmitted between sheep and goats [7].

LSD has been reported in a number of regions of Africa, where it is endemic, in the Middle East, and more recently in parts of Europe. There is a potential risk that LSDV could spread further into Europe and eventually worldwide [8–10]. In Eastern Africa, LSD was first reported in Kenya in 1957 [11], Sudan in 1972, and in Somalia in 1983 [12, 13]. There is no published literature about when LSD was first identified in Uganda, however the disease is thought to have spread from Southern Africa into Uganda between 1955 and 1960 [12]. The disease is currently present in all geographical regions of the country, with several outbreaks reported annually.

Outbreaks of LSD tend to be sporadic, and are likely dependent on animal movements, immune status of animals, and changes in weather patterns which affect vector populations. The main mode of transmission of LSDV is by mechanical arthropod vectors, such as biting flies, *Aedes aegypti* mosquitoes and three tick species belonging to the family Ixodidae (*Rhipicephalus (Boophilus) decoloratus, R. appendiculatus and Amblyomma hebraeum*) [14]. Predators, vermin and wild birds might also act as mechanical carriers of the virus [15, 16]. The virus can also be transmitted by fomites, such as equipment, clothing, and personnel [16]. Epidemics of LSD in non-endemic regions are reported to be associated with hot and wet seasons, as well as areas close to water bodies, swamps and marshlands that are conducive for breeding and multiplication of insects [17]. Spread of LSDV between farms and districts might be due to the lack of complete restriction of animal movements [18, 19].

LSD is known to cause substantial economic losses in the form of severe emaciation, lowered milk production, abortion, secondary mastitis, loss of fertility, extensive damage to hides leading to low quality of leather and loss of draught power from lameness [13, 20]. The morbidity rate in cattle can vary from 3 to 85% depending on the presence of insect vectors and host susceptibility. Mortality usually ranges between 1 to 5% [2], but can occasionally be as high as 20 to 85%. The disease is therefore a serious threat to the cattle farming community in endemic areas and is associated with trade restrictions following outbreaks. The livestock sector is one of Uganda’s important growth sectors contributing about US $290 million to the total GDP. Livestock constitutes 17% of the agricultural GDP and is a source of livelihood to about 4.5 million people in the country [21]. Trade in cattle hides generates about US $17 million annually and has potential for continued growth if conditions like LSD, which lower the quality of hides and skins, can be managed [22].

The success of any disease control program depends on a clear understanding of the epidemiology of the disease [23]. This requires analysis of available data to understand the distribution and patterns of spread of the disease [24]. Little has been studied about the epidemiology of LSD in Uganda, yet cattle farmers, district veterinary authorities, and monthly surveillance reports indicate the presence and impact of the disease in the country. Therefore, this study was conducted to describe the temporal and spatial distribution of reported outbreaks of LSD from 2002 to 2016 and to generate baseline epidemiological information on LSD in Uganda, which will facilitate further studies on disease prevalence and risk factors.

## Methods

### Study area

**Fig. 1:**
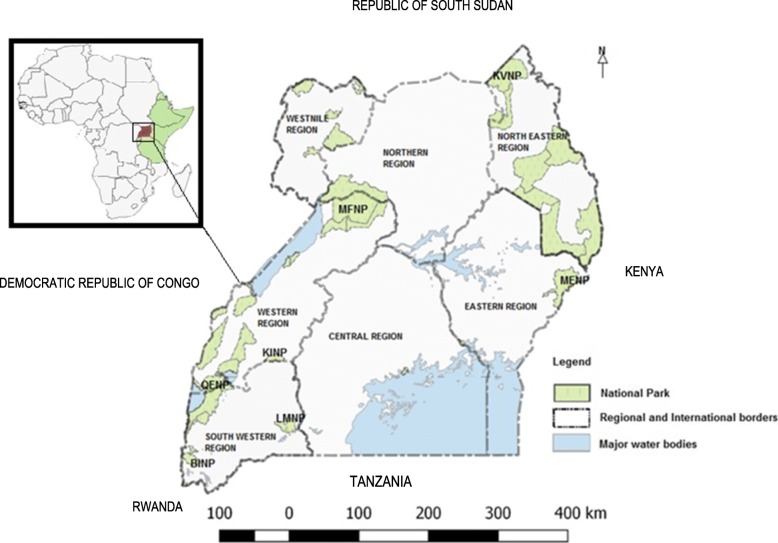
Map of Uganda showing the location of Uganda in Africa (inset), national parks, international borders and the regions in this study (Source: This study)

Uganda is a landlocked country located on the East African Plateau; it lies between latitudes 4°N and , and longitudes 29°35°E, with an area of about 241,038 km^2^. It is bordered by Kenya to the east, the Democratic Republic of the Congo to the west, South Sudan to the north, and Rwanda and Tanzania to the south. The southern part of the country includes a considerable portion of Lake Victoria, which is shared with Kenya and Tanzania (Fig. 1). Uganda lies within the Nile basin as well as the African Great lakes region, and has a diverse but generally equatorial climate. It is on average about 1100 m (3,609 ft) above sea level. Currently, the country is divided into 112 districts; each district is sub-divided into counties and sub-counties; each sub-county consists of several parishes and villages. Uganda has a number of national parks, however the major parks considered in this study are: Bwindi Impenetrable National Park (BINP), Kibale National Park (KINP), Kidepo Valley National Park (KVNP), Lake Mburo National Park (LMNP), Mount Elgon National Park (MENP), Murchison Falls National Park (MFNP), and Queen Elizabeth National Park (QENP).

### Data source and collection

Retrospective data on LSD outbreaks in Uganda during 2002–2016 were retrieved from the Ministry of Agriculture Animal Industry and Fisheries (MAAIF), Uganda. This information is based on the monthly disease surveillance reports submitted to MAAIF by District Veterinary Officers (DVOs). For the period considered in this study, Uganda had a varying number of districts (56–112). Reporting of LSD outbreaks was at the district level, thus making it impossible to disaggregate data from earlier years (56 districts) into the present 112 districts. Thus, all the data analysed here were aggregated into 56 districts consistent with 2002 boundaries. Districts were classified as adjacent to international borders and national parks. For data handling and presentation, districts were also grouped into seven geographical agro-ecological regions, which typically vary by rainfall and farming production systems (Fig. 1) [25]. Data on livestock numbers were obtained from the Uganda National livestock census report 2009 [26], and used to calculate cattle density.

In this paper, a case was defined as an animal with clinical signs or nodular lesions characteristic of LSD (with or without laboratory confirmatory diagnosis). An outbreak was defined as the occurrence of one or more cases of LSD in a particular herd. However, cases from nearby herds with frequent animal contact or shared grazing areas were also considered part of the same outbreak. New outbreaks were defined as those occurring in a herd separated from other herds by a fence or physical barrier such as hills, water bodies, forests or mountains. Each outbreak report contained data on the number of affected animals, susceptible animals, vaccinated animals, and deaths. Since the exact locations of the affected herds were not recorded, geographic coordinates of each district were defined as the centroid of the district.

### Data analysis

Descriptive analysis was performed on outbreaks, cases, and vaccination data. Count data on number of outbreaks and cases were tested for normal distribution using Shapiro-Wilk test and qqplots and found to be over-dispersed and positively skewed, with variance much larger than the mean. Therefore, Kruskal-wallis chi-squared tests and post-hoc Dunn’s tests with Bonferroni corrections for *p*-values were carried out to assess whether the differences in number of outbreaks between regions, months and years were statistically significant. Spearman’s correlation was used to investigate the relationship between cattle density and number of outbreaks and cases. QGIS version 2.18.9 with GRASS 7.2.1 [27] was used to plot the distribution of LSD outbreaks per district (2002–2016) and to create maps of the spatial and temporal distribution of LSD in Uganda. Software used for data analysis were Microsoft Office Excel, 2013 and R version 3.4.2.

Purely spatial, purely temporal, and space-time scan statistical analyses were performed using SaTScan™ v9.4.4 [28, 29]. The purely spatial scan statistic imposes a circular window of varying size upon the locations of possible outbreaks. The space-time scan statistic utilizes a dynamic cylindrical window, with a circular geographic base and with height corresponding to time. The purely temporal scan statistic uses a window with varying height corresponding to time, in the same way the height of the cylinder is used in the space-time scan. For each scan, the number of outbreaks in the window is recorded and compared to the null hypothesis of a random Poisson distribution, accounting for population size. A relative risk is calculated as the number of observed outbreaks within a window divided by the number of expected outbreaks across the study area. The window with the maximum log likelihood ratio (LLR) is defined as the most likely cluster. LLR is calculated by

$$ LLR=\log {\left(\frac{n}{E(n)}\right)}^2{\left(\frac{N-n}{N-E(n)}\right)}^{\left(N-n\right)}{I}^{\prime } $$

where N is the total number of cases; n is the observed number of cases within the scan window; *E(n)* and *N – E(n)* are the expected number of cases within and outside the window under the null hypothesis, respectively, and *I* is an indicator function (equal to 1 when the window has more cases than expected under the null hypothesis and 0 otherwise). Here, scans were conducted for areas of high rates, testing for elevated risk within a window as compared to outside. District centroids were tested as potential outbreak locations, and the maximum possible spatial and/or temporal cluster size was set to 50% of the total population at risk. Monte Carlo simulation (*n* = 999 permutations) was used to determine the significance of detected clusters [30].

## Results

A total of 1161 LSD outbreaks were reported at the district level from January 1, 2002 to December 31, 2016, with an average of 77 (± 51.4 SD) outbreaks per year and a median of 70 outbreaks per year. During this 15-year period, 319,552 cases were recorded, with an average of 21,303 ± 4121 SD cases per year, and 2169 recorded deaths (average of 146 ± 17 SD deaths per year) attributed to LSD. Morbidity, mortality and case fatality rates were 4.77, 0.03 and 0.72%, respectively (Table 1).

**Table 1:** Average annual number of outbreaks, morbidity, mortality and case fatality rates in different regions of Uganda. Population at risk refers to the number of susceptible cattle in herds where at least one case was reported

Region	No. of Outbreaks	Population at Risk	No. of Sick	No. of Dead	Morbidity rate (%)	Mortality rate (%)	Case fatality rate (%)
Central	28	306,452	5746	49	1.88	0.02	0.86
East	25	75,323	12,112	16	16.08	0.02	0.13
North	9	18,878	1440	15	7.63	0.08	1.02
North East	2	268	61	2	22.90	0.87	3.80
South West	9	27,786	1228	24	4.42	0.09	1.95
West	3	17,222	639	43	3.71	0.25	6.69
West Nile	1	647	77	4	11.97	0.64	5.34
Total	77	446,575	21,303	153	4.77	0.03	0.72

### Spatial distribution of LSD

**Fig. 2:**
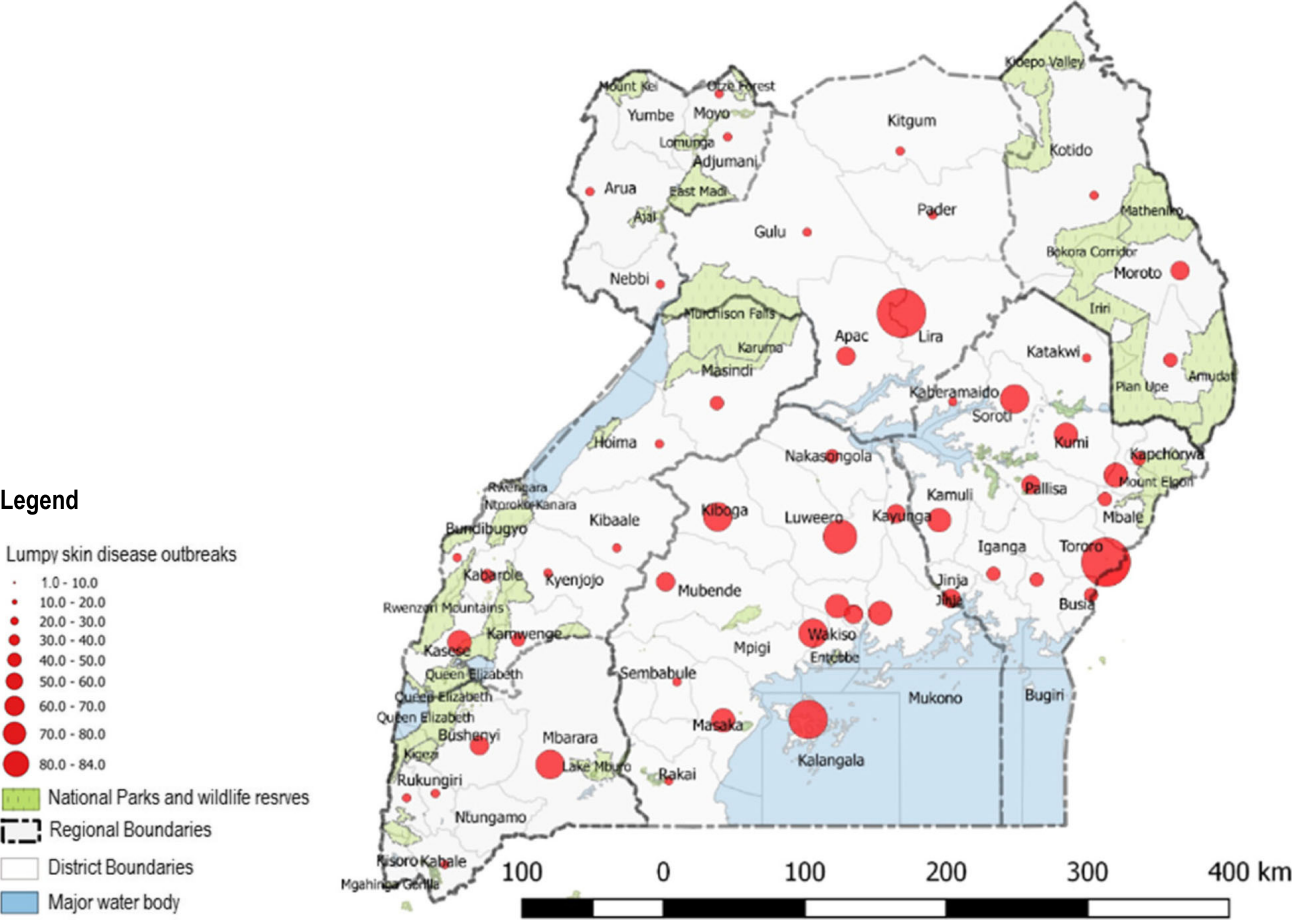
Map of Uganda showing district and regional distribution of LSD outbreaks (2002–2016), national parks and national borders. The size of the red circles indicate the respective number of LSD outbreaks in the areas marked (Source: This study)

The distribution of LSD at the district and regional level was mapped (Fig. 2) to represent the spatial pattern of outbreaks (2002–2016). The disease was reported in 55 out of 56 districts during this period. Lira (*n* = 84, 6.3%) and Tororo (*n* = 83, 6.3%) had the highest number of outbreaks (2002–2016) while Kisoro (*n* = 1, 0.075%), Mayuge (*n =* 1, 0.075%) and Ntungamo (*n =* 1, 0.075%) had the lowest numbers of outbreaks during the period studied. No LSD outbreaks were reported in Yumbe district. There was a significant difference between the numbers of outbreaks by region (*P* < 0.002), with the Central region (*n* = 418, 36%) reporting the highest number of outbreaks followed by the Eastern region (*n* = 372, 32%), Southwestern region (*n* = 140, 12%), Northern region (*n* = 131, 11%), Western region (*n =* 41, 4%), Northeastern region (*n =* 37, 3%), and Northwestern (*n* = 22, 2%) region. An additional file shows this in more detail [see additional file 1]. We found significant differences in number of outbreaks by region for the following pairs of regions; Central-West (Dunn’s test, *p* = 0.004), Central-West Nile (Dunn’s test, *p* = 0.013), North-West (Dunn’s test, *p* = 0.02), and North-West Nile (Dunn’s test, p = 0.02). Spearman’s correlation showed a significant correlation between cattle density and number of outbreaks, and no significant correlation between cattle density and cases respectively (r_s_ = 0.27, *p*-value = 0.04; r_s_ = 0.12, p-value = 0.37 respectively). Seventeen (17) out of 56 districts adjacent to national parks reported only 45 (3.9%) outbreaks, while 332 (28.6%) outbreaks were reported in districts adjacent to international borders. When outbreaks in districts adjacent to national parks were compared according to which national park they bordered, we observed that districts bordering Queen Elizabeth National Park (QENP) reported a higher number of outbreaks than those reported by districts bordering the other six national parks (an additional file shows this in more detail [see additional file 2]).

### Incidence of LSD outbreaks adjacent to the international Borders

The 22 of 56 districts adjacent to the international borders reported 332 (28.6%) LSD outbreaks as compared to 829 (71.4%) outbreaks from districts with no international border. The number of LSD outbreaks varied between the different international borders, the highest being adjacent with Kenya (157 outbreaks in 6 districts) and DRC borders (87 outbreaks in 7 districts), while 55 outbreaks were reported in 2 districts bordering Tanzania and 21 outbreaks were reported in 4 districts bordering South Sudan. The lowest number of LSD outbreaks was reported among the districts bordering Rwanda (12 outbreaks in 3 districts). Analysis of these differences by Kruskal Wallis test however revealed no significant difference in the numbers of outbreaks per international border (p-value = 0.41).

### Temporal distribution of LSD

**Fig. 3:**
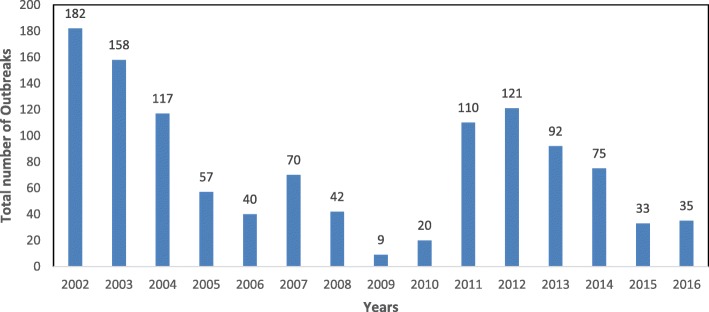
Total yearly Lumpy skin disease outbreaks in Uganda from 2002 to 2016

**Fig. 4:**
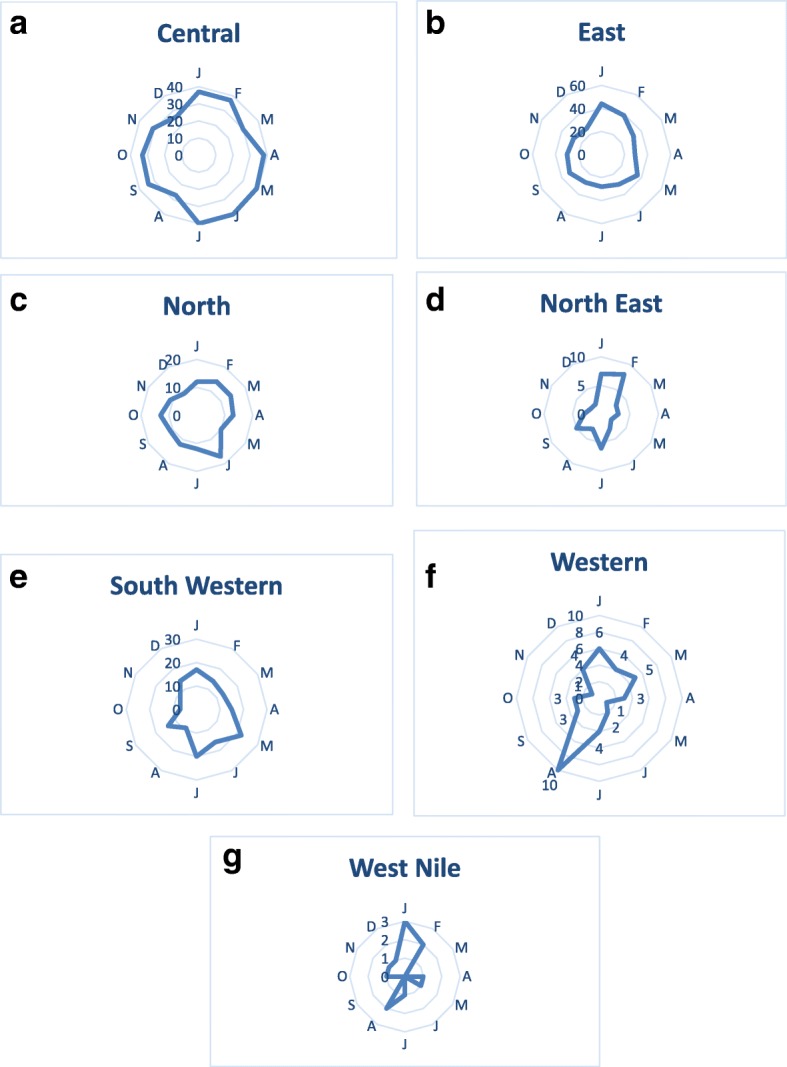
Spider plots showing the monthly distribution of LSD outbreaks per region from 2002 to 2016

On average, 22 districts (± 9.8 SD) experienced outbreaks of LSD each year. High annual incidences of LSD outbreaks were reported in 2002 (*n* = 182 outbreaks), 2003 (*n* = 153), 2004 (*n* = 117), 2011 (*n* = 110) and 2012 (*n* = 121) while the lowest annual incidence was reported in 2009 (*n* = 9) (Fig. 3). The highest incidence was reported in the month of January (*n =* 117 across all years), which accounted for 10% of all outbreaks reported, and the lowest in November (*n* = 80), accounting for 6.9% of all reported outbreaks. There was no significant difference in the incidence of outbreaks between months (*p* = 0.443). When the overall data were grouped into four seasons, two wet seasons and two dry seasons, the highest incidence was reported in the first dry season (Dec–Feb, *n* = 312, 26.9%) followed by second dry season (Jun–Aug, *n* = 300, 25.8%), first wet season (Mar–May, *n* = 286, 24.6%) and second wet season which had the lowest incidence (Sep-Nov, *n* = 263, 22.7%). More marked intra-annual variation was observed when subdividing the analysis by region, with the northeastern (Fig. 4d), western (Fig. 4f), and West Nile region showing more profound seasonal patterns (Fig. 4g).

### Purely spatial clusters of lumpy skin disease

**Fig. 5:**
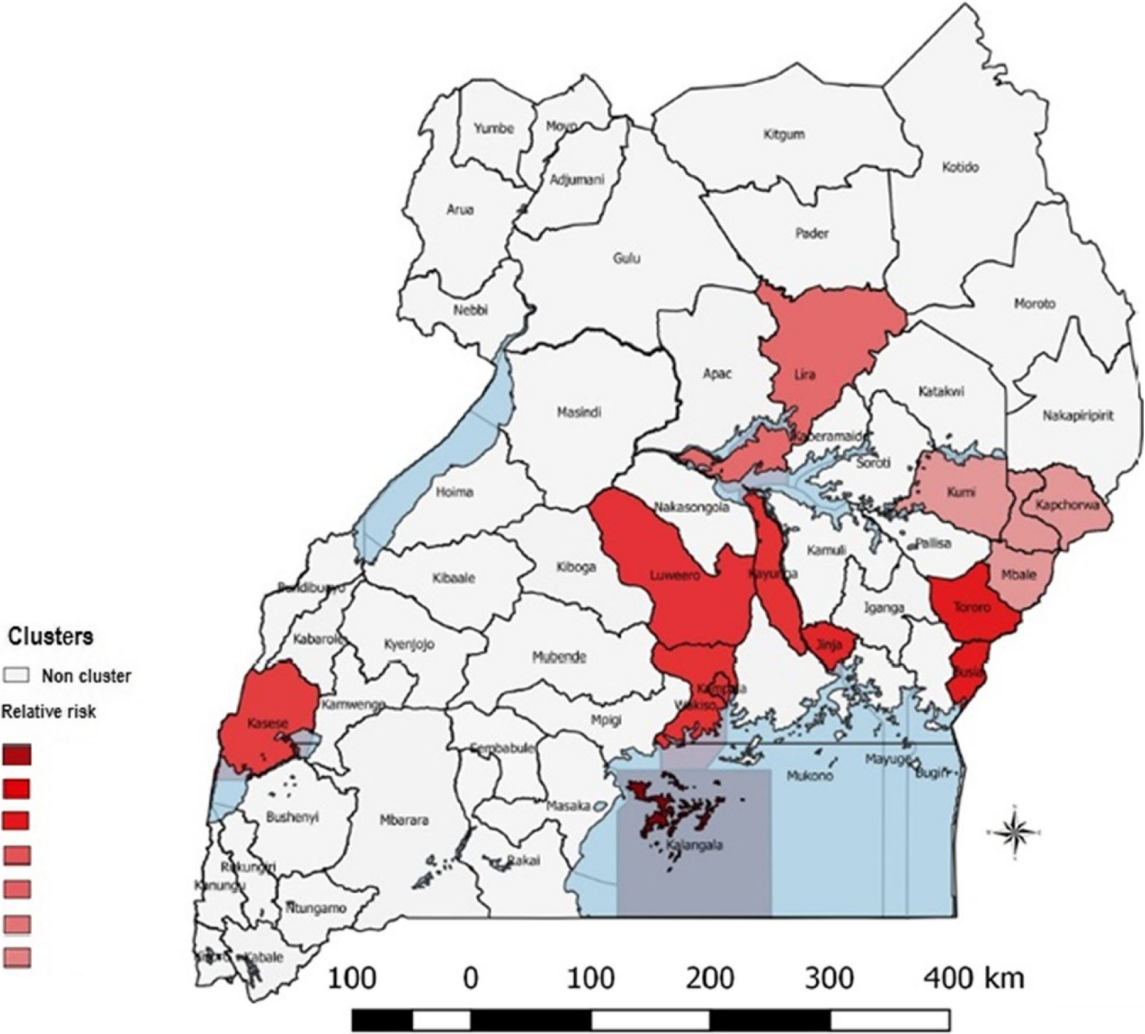
Purely spatial distribution of identified clusters of LSD cases with significantly higher incidences in Uganda from 2002 to 2016 (Source: This study)

The spatial pattern of LSD was found to be nonrandom. A total of 7 clusters were identified, two (2) of which were located in Central region, three (3) in Eastern region, one (1) in Southwestern region and one (1) in Northern region (Fig. 5). The most likely cluster was observed in the Kalangala district in Central Uganda. The radius of the cluster was 0 km, indicating that the cluster only included Kalangala district. The relative risk (RR) was 156.17, indicating that cattle within this district were around 156 times more likely to be affected by LSD than in areas outside the cluster (Table 2). The observed number of outbreaks for this cluster was 66 compared with a calculated 0.45 expected outbreaks. Secondary clusters were located in (Luwero, Kayunga, Wakiso, and Kampala), found in central Uganda; (Busia, Tororo), Jinja, (Kapchorwa, Sironko, Mbale and Kumi) in Eastern Uganda; Kasese in Southwestern Uganda; and Lira in Northern Uganda; (Table 2 and Fig. 5). RR for these clusters ranged from just over 1.8 to over 9.

**Table 2:** SaTScan statistics for purely spatial clusters with significantly higher incidence of LSD in Uganda from 2002 to 2016

District/location	Coordinates/radius	Number of outbreaks	Expected outbreaks	Relative risk	Log likelihood ratio	*P*-value
Kalangala	(0.320837 S, 32.293743 E) / 0 km	66	0.45	56.17	265.81	< 10^−17^
Busia, Tororo	(0.470669 N, 34.091980 E) / 25.35 km	103	11.30	9.93	139.83	< 10^− 17^
Luwero, Kayunga, Wakiso, Kampala	(0.840409 N, 32.497668 E) / 55.57 km	135	30.46	4.90	101.58	< 10^−17^
Kasese	(0.169899 N, 30.078078 E) / 0 km	34	7.50	4.64	25.19	< 2.3 × 10^−10^
Jinja	(0.447857 N, 33.202612 E) / 0 km	20	3.11	6.54	20.48	< 2.5 × 10^−8^
Lira	(2.258083 N, 32.887407 E) / 0 km	95	49.24	2.01	17.65	< 4.2 × 10^−6^
Kapchorwa, Sironko, Mbale, Kumi	(1.335021 N, 34.397636 E) / 54.24 km	94	52.18	1.87	14.33	< 1.1 × 10^−5^

### Space-time clusters of lumpy skin disease

**Fig. 6:**
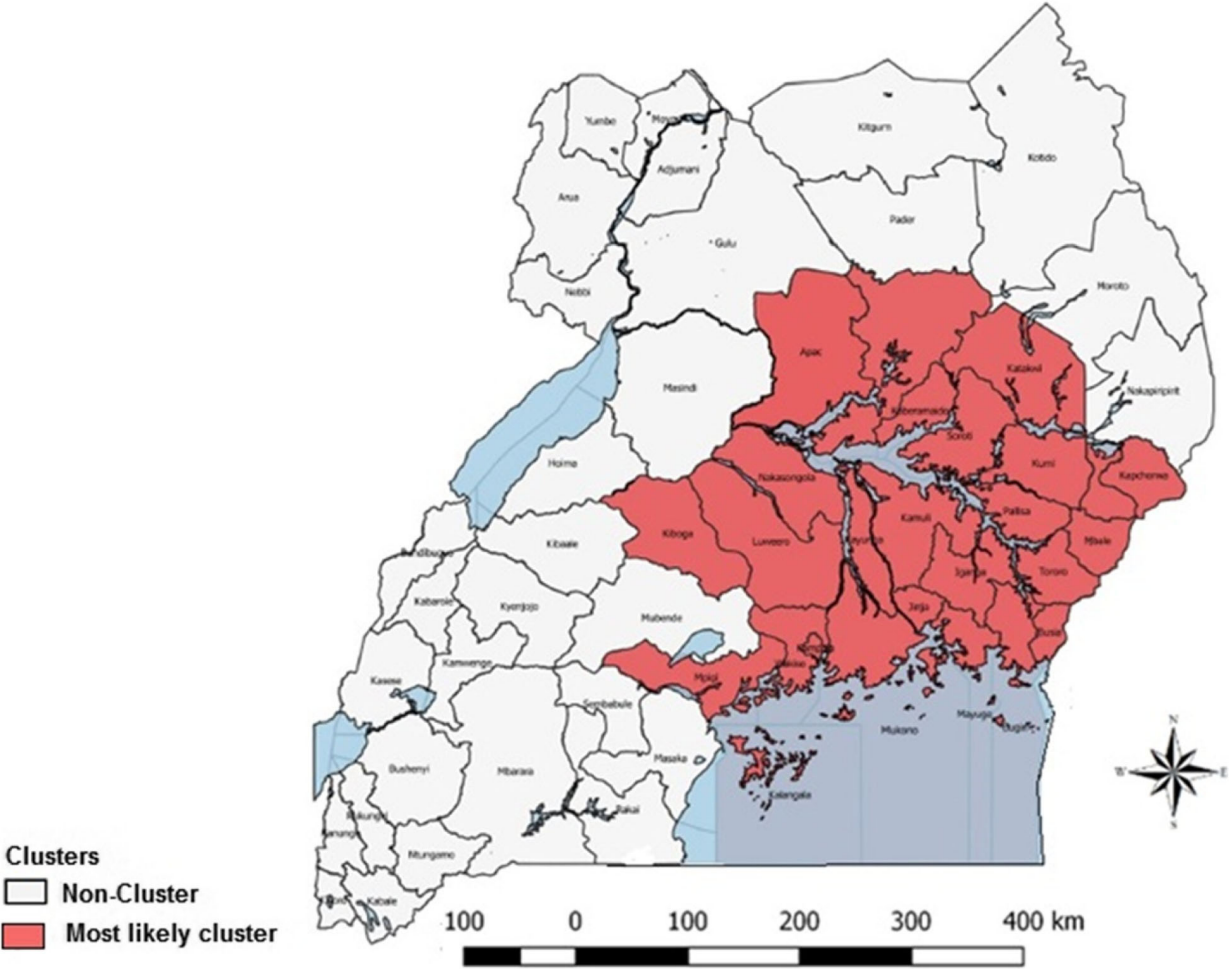
Space-time distribution of identified clusters (*n* = 5) of LSD cases with significantly higher incidences in Uganda from 2002 to 2016 (Source: This study)

One space-time cluster was identified and it persisted for a duration of 3 years. This space-time cluster was located in 24 districts found in Eastern and Central region, and in 2 districts found in Northern region. This space-time cluster was from January 1, 2002 - December 31, 2005, with 383 observed outbreaks, compared to a calculated 137.97 expected outbreaks. The space-time cluster is shown in Table 3 and Fig. 6.

**Table 3:** SaTScan statistics for a space-time cluster with a significantly higher incidence of LSD in Uganda from 2002 to 2016

District/location	Coordinates/radius	Timeframe	Number of outbreaks	Expected outbreaks	Relative risk	Log likelihood ratio	*P*-value
Kamuli, Kayunga, Iganga, Jinja, Pallisa, Luwero, Mukono, Bugiri, Kaberamaido, Nakasongola, Kampala, Mayuge, Wakiso, Soroti, Kumi, Mbale, Busia, Mpigi, Tororo, Sironko, Apac, Kapchorwa, Lira, Katakwi, Kiboga, Kalangala	(0.944785 N, 33.126717 E) / 168.37 km	2002.1.1 to 2005.12.31	383	137.97	3.69	179.08	< 10^−18^

### Purely temporal clusters of LSD

Temporal cluster analysis of LSD outbreaks in Uganda showed one peak period with only one cluster identified during January 1, 2002 to December 31, 2004. The overall RR within the cluster was 2.34 (LLR =85.92, *P* = 0.001) with 417 observed outbreaks compared to 226.24 expected outbreaks.

## Discussion

To understand the spatial epidemiology of lumpy skin disease (LSD) outbreaks in Uganda, we described the geographic and temporal occurrence of LSD and analyzed the data for spatial and temporal clusters using retrospective data collected between 2002 and 2016. During this period, an average of 77 LSD outbreaks were reported across 22 (±9.8 SD) districts each year, demonstrating that LSD is endemic in Uganda.

Incidence of reported LSD outbreaks differed between regions, with more outbreaks reported in the Central and Eastern regions as compared to the rest of the regions. The Central and Eastern regions represented more than half of the reported LSD outbreaks during this time-frame. This marked difference could be due to a number of factors including animal husbandry practices, presence of high numbers of insect vectors, higher frequency of exotic cattle breeds, awareness of disease control, uncontrolled animal movements, and potentially biases in disease reporting related to proximity to the central administrative center of MAAIF in Kampala [24, 31, 32]. However, the primary contributor to the high rates of reported outbreaks in this region of Uganda may be climate, given that the Central and Eastern regions of Uganda form part of the Lake Victoria basin; these two regions also have other lakes (Kyoga, Opeta, and Bisina), rivers (Nile, Manafwa, Mpologoma, Malaba) and wetlands which provide wet and humid micro-climates [33, 34]. This, coupled with an average monthly temperature range of 22 °C–29 °C, provides suitable conditions for multiplication of arthropod vectors for LSD [14]. The only published studies about arthropod vectors in this region are studies reporting distribution of *Glossina spp,* [35, 36] which are known to transmit LSD mechanically [37]. These studies have found high density of these flies in the Central and Eastern regions of Uganda, thus suggesting that the climatic conditions are suitable for arthropod vector multiplication and survival.

Spatial, temporal and space-time scan statistics are tools used to detect aggregations of disease outbreaks or cases and identify whether these outbreaks or cases of disease in space or time can be explained by chance alone or are statistically significant. Clusters may occur due to local transmission of the disease or due to shared risk factors within an area. We investigated the spatial distribution of LSD outbreaks and identified areas with high endemicity of LSD and clustering patterns using spatial scan statistics. We showed that in the period from 2002 to 2016 as a whole, the geographic distribution patterns of LSD outbreaks in Uganda were not random. Spatial cluster analysis identified 7 clusters, which were primarily located in the Central and Eastern regions. The most likely spatial cluster was observed in Kalangala district in Central Uganda. High incidence in this region is likely driven by climate and presence of wetlands in this district. Kalangala is a district made up of 84 islands surrounded by Lake Victoria, with 95% of the district area covered by water bodies, and mean annual rainfall ranging from 1125 to 2250 mm [38]. The climate of this district is generally moist and humid all through the year with moderately small seasonal variations of temperature, humidity, and wind throughout the year [38]. These conditions are known to maintain arthropod vectors which transmit LSD. District local government reports list Lumpy skin disease among the most economically important Livestock diseases in the district [38], which is in agreement with our findings. Similar factors may also play a role in creating the other hotspots identified in the spatial cluster analysis.

We also conducted a space-time cluster analysis in addition to the purely spatial cluster analysis. A single space-time cluster was identified. When we compared the results of the purely spatial cluster analysis with those of the space-time cluster analysis, we found that thirteen of fourteen districts, identified as purely spatial clusters, were also identified as part of the space-time cluster. However, one district (Kasese) identified by the purely spatial analysis did not appear in the space-time cluster. The districts in the space-time cluster were found in Central, East and Northern parts of the country, and the duration of the associated space-time cluster was for four years. These areas thus appear to have experienced an epidemic wave of LSD for this four year period, occurring mainly within the endemic hotspots.

We found that LSD occurs throughout the year with outbreaks reported every month. In the more endemic areas around the Lake Victoria and Lake Kyoga basin of Uganda, there is rainfall throughout most of the year, providing hot and wet weather conditions which are conducive for breeding of biting flies which are known to transmit LSD. During the dry season (December to February), there is reduced availability of pasture and water, so cattle are moved to swampy marsh lands which are common in Central and Eastern regions of the country and present in the other regions as well. These swampy areas maintain a hot and wet micro-climate which support large populations of biting insects; this together with a surge in cattle herds competing for limited grazing areas may lead to spread of LSD and therefore an increase in the number of new outbreaks reported. The Central and Eastern regions showed no seasonal pattern of LSD outbreaks, however the Northeast, West Nile and West showed more seasonal patterns of outbreaks. A slight increase in the number of outbreaks was observed around the month of August for the West and West Nile regions, and an increase in outbreaks was observed in January and February for the Northeast and West Nile regions (Fig. 4). This suggests that seasonal factors have greater effects on incidence for these regions. These results further substantiate the suggestion that the epidemiology of LSD may differ in the endemic hotspots of Central and Eastern Uganda, characterized by less seasonality, presence of spatial clusters as well as space-time clusters of outbreaks, and non-endemic zones that experience sporadic outbreaks but no persistent circulation. Interestingly, endemic hotspots (Central and Eastern) had strikingly lower mortality and case-fatality rates than the other regions, which further suggests an underlying difference in the disease’s epidemiology and impact in these different zones. However, management and ecological factors could also impact the fatality rate of the disease.

There were two evident temporal waves of LSD spread during which high number of outbreaks were reported, spaced about ten years apart (Fig. 3). Temporal cluster analysis also identified the first of these two temporal waves, January 2002 to December 2004 as a period with heightened occurrence of LSD in Uganda. Low numbers of outbreaks were reported in 2009, but we do not have sufficient data to propose factors responsible for this occurrence.

Uganda has seven (7) major game parks; these parks are not fenced and it is therefore common for livestock to graze with wildlife. While there have been few studies elsewhere in Africa investigating the role of wildlife in the transmission of LSD, the 17 districts bordering national parks accounted for only 3.9% (*n* = 45) of the total LSD cases reported. However, parks may vary in terms of the types of wildlife species present and the extent to which wildlife and livestock interact. It is notable that 20 out of the total 45 outbreaks bordering national parks (44.4%) were reported in districts bordering Queen Elizabeth National Park (QENP). QENP holds populations of African Buffalo (*Syncerus caffer*), kudu and waterbuck, which have previously been shown to have antibodies against LSDV [6, 39, 40] and therefore could be potential hosts for the virus. It must however be noted that three (3) other parks are inhabited by buffaloes, and the extent of wildlife-livestock interaction may vary in these parks thus limiting cross species transmission of LSD. Neutralizing antibodies have previously been detected in African Buffalo sera from QENP [39]. Though this was in the 1980s, these findings suggest that wildlife may play a role in the maintenance cycle of LSD. More research is needed to clarify the role of buffalo. Genotyping of LSD at wildlife-livestock interfaces, as well as at international borders, should be performed to determine the molecular epidemiology of the disease and shed more light on the effect of wildlife and cross-border animal movements. When we compared outbreaks in districts adjacent to international borders, we found that even if 47.3% of these 332 outbreaks were reported at the Kenya-Uganda border, this difference was found not statistically significant when compared with outbreaks from the four other international borders of Uganda.

The findings of this study should be interpreted with caution because of the potential bias related to underreporting of outbreaks and cases [41]. In addition, the cases were determined based on clinical signs with no confirmatory diagnostic tests, which may have led to biases occurring from nonreporting of sub-clinical cases. Outbreak location information was at the district level, which therefore prevented more elegant spatial analyses and made it difficult to more precisely assess the role of spatial proximity to international boundaries or national parks. More purposeful sampling schemes based on active surveillance and molecular epidemiology are needed to better resolve risk factors and dynamics of LSD spread in Uganda.

## Conclusions

Uganda’s hot and wet climate provides a conducive environment for biting arthropods which are known to transmit LSD. In this study, we demonstrate that LSD is endemic in Uganda, with annual outbreaks in all regions of the country, albeit in varying incidence. We identified potential endemic hotspots for LSD outbreaks, highlighting the need for risk-based surveillance in these areas to establish the actual disease prevalence and risk factors for maintenance of the disease. Our space-time analysis also revealed that sporadic LSD outbreaks tend to occur within endemic hotspot areas. Interestingly, endemic hotspots had less seasonality in incidence and strikingly lower mortality and case-fatality rates than the other regions, suggesting that epidemiology and impact of LSD may vary within and outside these hotspots. Based on our findings, we suggest that true prevalence of the disease, and viral genotypes, should be determined in order to inform appropriate control measures in these endemic hotspots, such as vaccination, to prevent further spread of the disease. LSD should be included amongst the priority cattle diseases in Uganda, where regular surveillance and vaccination are done by the government. Our findings provide a baseline for further studies into the epidemiology of LSD in Uganda and East Africa.

## Additional files

Additional file 1: Mean annual Lumpy skin disease outbreaks across different regions (agro-ecological zones) from 2002 to 2016. The mean annual Lumpy skin disease outbreaks reported in the Central, East, North, Northeast, Southwest, West and Westnile regions of Uganda from 2002 to 2016. (DOCX 33 kb)

Additional file 2: Occurrence of LSD outbreaks in districts adjacent to national parks in Uganda 2002–2016. This table shows the yearly number of Lumpy skin disease outbreaks reported in districts bordering each of the seven major national parks in Uganda. A total of forty five outbreaks were reported, notably twenty out of these forty five outbreaks are from districts bordering Queen Elizabeth national park. (DOCX 13 kb)

## Abbreviations

BINP: Bwindi Impenetrable National Park
DVO: District Veterinary Officer
KINP: Kibale National Park
KVNP: Kidepo Valley National Park
LMNP: Lake Mburo National Park
LSD: Lumpy Skin Disease
MAAIF: Ministry of Agriculture Animal Industry and Fisheries
MENP: Mount Elgon National Park
MFNP: Murchison Falls National Park
QENP: Queen Elizabeth National Park
