# Supplementary figures and images for: Spatial and temporal distribution of lumpy skin disease outbreaks in Uganda (2002–2016)
Source: BMC Vet Res. 2018 Jun 1;14:174. doi: 10.1186/s12917-018-1503-3 (PMC5984736; doi:10.1186/s12917-018-1503-3)

**
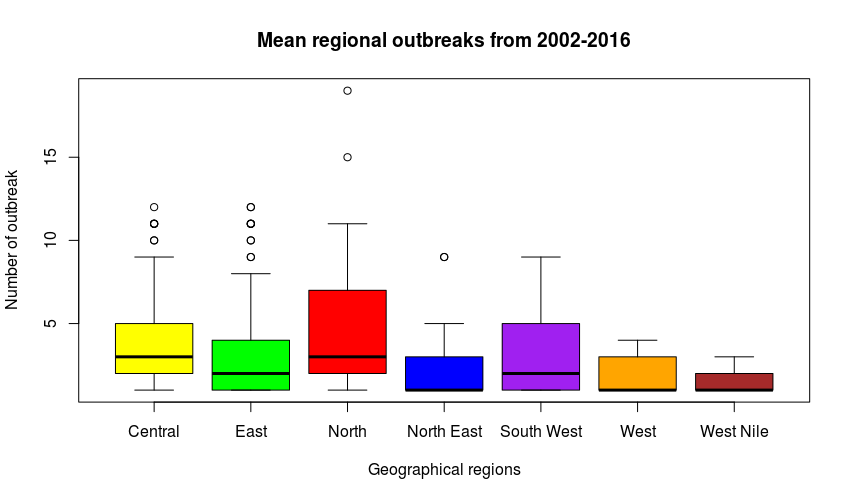
**

Supplement: Supplementary file 1 — Mean annual Lumpy skin disease outbreaks across different regions (agro-ecological zones) from 2002 to 2016. The mean annual Lumpy skin disease outbreaks reported in the Central, East, North, Northeast, Southwest, West and Westnile regions of Uganda from 2002 to 2016. (DOCX 33 kb) [file 12917_2018_1503_MOESM1_ESM.docx]
