# Supplementary material for: Spatial and temporal distribution of lumpy skin disease outbreaks in Uganda (2002–2016)
Source: BMC Vet Res. 2018 Jun 1;14:174. doi: 10.1186/s12917-018-1503-3 (PMC5984736; doi:10.1186/s12917-018-1503-3)
Supplement: Supplementary file 2 — Occurrence of LSD outbreaks in districts adjacent to national parks in Uganda 2002–2016. This table shows the yearly number of Lumpy skin disease outbreaks reported in districts bordering each of the seven major national parks in Uganda. A total of forty five outbreaks were reported, notably twenty out of these forty five outbreaks are from districts bordering Queen Elizabeth national park. (DOCX 13 kb) [file 12917_2018_1503_MOESM2_ESM.docx]

|  | Year of occurrence | | | | | | | | | | | | | | | |  | |
| --- | --- | --- | --- | --- | --- | --- | --- | --- | --- | --- | --- | --- | --- | --- | --- | --- | --- | --- |
| National Park | **2002** | **2003** | **2004** | **2005** | **2006** | **2007** | **2008** | **2009** | **2010** | **2011** | **2012** | **2013** | **2014** | **2015** | **2016** | **Total** | |  |
| BINP | 1 | - | - | - | - | - | 1 | - | - | - | - | - | - | - | - | 2 | |  |
| KINP | 1 | 1 | 1 | - | 1 | 2 | - | - | - | 1 | 1 | - | 1 | - | - | 9 | |  |
| KVNP | - | - | - | - | - | - | - | - | - | - | - | - | - | - | - | 0 | |  |
| LMNP | - | - | - | - | - | - | - | - | - | - | 1 | - | 1 | 1 | 1 | 4 | |  |
| MENP | - | 1 | 1 | - | - | - | - | - | 1 | 1 | - | - | - | 1 | - | 5 | |  |
| MFNP | 1 | 1 | - | - | - | - | - | - | 2 | - | - | - | - | - | 1 | 5 | |  |
| QENP | 2 | 3 | 2 | 0 | 1 | 2 | 1 | 1 | 1 | 1 | 1 | 2 | 1 | 1 | 1 | 20 | |  |

BINP: Bwindi impenetrable national park, KINP: Kibale national park, KVNP: Kidepo valley national park, LMNP: Lake Mburo national park,

MENP: Mount Elgon national park, MFNP: Murchison falls national park, QENP: Queen Elizabeth national park
